# Development of a soil cadmium bioaccessibility prediction model for human health risk assessment

**DOI:** 10.1016/j.isci.2026.115679

**Published:** 2026-04-11

**Authors:** Jianghao Cao, Jie Xiong, Chaoyan Zhang, Ying Zhao, Xiaoxin Guo, Yimin Sang, Heming Wang, Youya Zhou

**Affiliations:** 1 Technical Centre for Soil, Agricultural and Rural Ecology and Environment, Ministry of Ecology and Environment, Beijing 100012, China; 2 College of Chemical Engineering and Environment, State Key Laboratory of Heavy Oil Processing, China University of Petroleum, Beijing 102249, China; 3 School of Mechanical Engineering, Beijing Institute of Petrochemical Technology, Beijing 102617, China

**Keywords:** Environmental science, Modeling in soil science, Soil science

## Abstract

Soil cadmium (Cd) contamination threatens human health, especially in industrial regions. Conventional risk assessments rely on total metal concentrations while neglecting bioaccessibility and exposure parameter uncertainty, potentially biasing estimates. This study compiled 195 datasets of soil properties and Cd bioaccessibility across China. Correlation analysis and random forest modeling were employed to identify key factors and develop predictive models for bioaccessible Cd content. Total Cd content was the dominant determinant, with soil pH, sand fraction, and iron content exerting additional effects. The random forest model exhibited excellent performance (test set coefficient of determination [R^2^] = 0.87). Nationwide predictions revealed a pronounced north-south contrast in Cd bioaccessibility (1.38%–55.60%). Incorporating predicted values into Monte Carlo-based probabilistic risk assessment reduced health risk estimates by 3.30–7.61 times, highlighting bioaccessibility as a major source of uncertainty, particularly for children. This model provides an effective tool for rapid prediction of soil Cd bioaccessibility and supports refined health risk assessment and management.

## Highlights

• Developed a random forest model for cadmium bioaccessibility in diverse soils
• Soil cadmium bioaccessibility shows a north-south gradient in China
• Bioaccessibility reduces risk overestimation and interregional variability
• Sensitivity analysis shows bioaccessibility is a key parameter

## Graphical abstract

**Figure:**
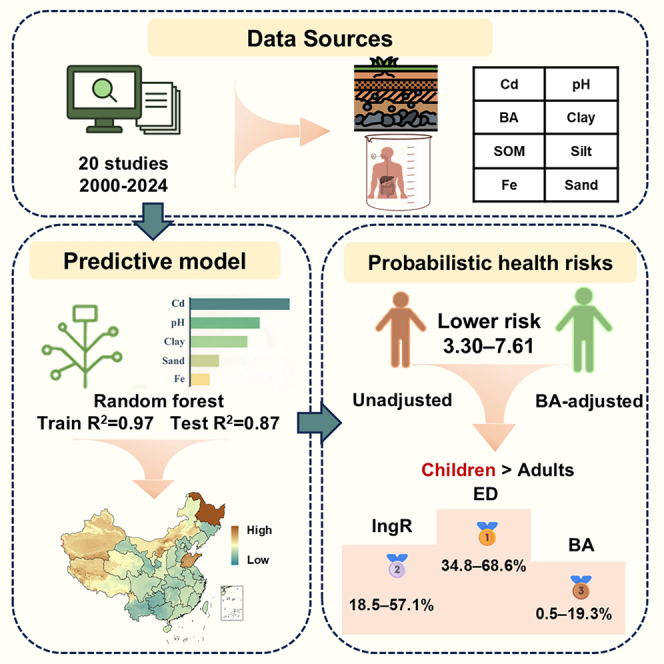

## Introduction

With the advancement of industrialization and urbanization, soil heavy metal contamination has become a global environmental and public health concern. Cadmium (Cd) exhibits high mobility and strong bioaccumulation potential, allowing it to accumulate in the human body and cause chronic health effects such as kidney damage and bone metabolism disorders. It is therefore considered one of the priority pollutants of concern.^1^ Global-scale soil pollution prediction studies have shown that, among various heavy metals, Cd has the highest exceedance rate, approximately 9%, highlighting its significance in global soil environmental management.^2^ In industrialized regions such as the United States and Europe, historical industrial and mining activities have left numerous contaminated sites where Cd accumulation is common.^3^ China faces similar challenges. Previous studies indicate that Cd is one of the most heavily contaminated and widely studied heavy metals at contaminated sites, with a spatial distribution pattern characterized by higher levels in the southeast and lower levels in the northwest.^4^ Incidental oral ingestion of soil represents an important exposure pathway for Cd, posing particularly significant health risks to children living near contaminated sites.^5^

Since only a fraction of soil Cd can be absorbed by the human body and enter systemic circulation, health risk assessments based solely on total concentrations of heavy metals may overestimate the actual health risks.^6^ In actual exposure scenarios, the parameter of concern is the bioaccessible concentration, defined as the maximum potential amount of a contaminant that can dissolve in simulated gastrointestinal fluids based on *in vitro* assays.^7^ The ratio of the bioaccessible concentration to the total concentration is referred to as bioaccessibility.^8^ Several countries, including China, have issued technical standards and guidelines to promote the incorporation of bioaccessibility testing into soil contamination risk assessment frameworks.^9^^,^^10^^,^^11^^,^^12^ However, Cd bioaccessibility is influenced by multiple soil properties,^13^ which often exhibit significant spatial heterogeneity across regions.^14^^,^^15^ For example, the sand content in Chinese soils ranges from 6.53% to 74.83%.^16^ Consequently, the bioaccessibility of different contaminated sites must be assessed individually, a time-consuming and expensive process. Developing a reliable predictive model could enable rapid estimation of Cd bioaccessibility in practical site applications, thereby effectively addressing this challenge.

Existing empirical models for predicting the bioaccessibility of soil heavy metals are typically constructed using multiple linear regression or stepwise regression approaches.^14^^,^^17^ However, such linear models are susceptible to outliers and may struggle to capture the complex, nonlinear interactions among soil properties. In recent years, machine learning methods have been increasingly applied in environmental science research,^18^ including algorithms such as random forest, extreme gradient boosting, support vector regression, and artificial neural networks. Among these approaches, the random forest model has notable advantages in handling nonlinear relationships and complex interactions among variables. It is also relatively insensitive to multicollinearity and exhibits strong fault tolerance. As a result, it has demonstrated stable and superior performance in environmental and health risk prediction studies.^19^^,^^20^^,^^21^ For example, Xiao et al.^22^ compared four machine learning algorithms for predicting the bioaccessibility of polycyclic aromatic hydrocarbons (PAHs) in food and found that the random forest model achieved the best predictive performance. In contrast, although neural networks are also suitable for nonlinear modeling, their performance is generally more dependent on the size and representativeness of the training dataset.^20^ Previous studies have attempted to apply machine learning methods to predict soil Cd bioaccessibility. For instance, Xie et al.^21^ developed a random forest model (coefficient of determination [R^2^] = 0.86) based on soil physicochemical properties and heavy metal speciation, demonstrating good predictive performance. However, the model was designed specifically for mining and smelting sites and was limited to gastric phase bioaccessibility, without considering the intestinal phase. Nevertheless, the intestinal phase represents the primary stage during which substances are absorbed into the human bloodstream.^23^

In addition to bioaccessibility, conventional health risk assessments that rely on deterministic parameter values may fail to accurately reflect actual exposure conditions, potentially leading to underestimation or overestimation of health risks.^24^^,^^25^ Probabilistic risk assessment incorporates parameter uncertainty into the evaluation framework, offering a feasible solution to this issue.^26^ In particular, the Monte Carlo simulation approach has been increasingly studied and applied to evaluate health risks.^27^ This method incorporates probability distributions of input parameters and performs extensive random sampling to generate probabilistic risk estimates and calculate different percentiles and exceedance probabilities. It also enables the quantitative evaluation of the contribution of each parameter to overall uncertainty, thereby improving the reliability of risk assessment.

In assessments of soil heavy metal-related health risks, the influences of bioaccessibility and parameter uncertainty are often overlooked.^25^^,^^28^ Therefore, the objectives of this study were to: 1) systematically integrate soil datasets from multiple regions and soil types across China to overcome the limitations associated with single-region or single-type samples, 2) identify the key soil factors influencing Cd bioaccessibility through correlation analysis and develop a reliable predictive model for intestinal-phase bioaccessible Cd content applicable to different geographic regions of China, and 3) conduct a probabilistic risk assessment of soil Cd contamination using Monte Carlo simulation based on the nationwide model-predicted results, in order to achieve a more precise quantification of health risks across China’s major geographical regions and provide scientific support for refined soil heavy metal risk assessment and environmental management.

## Results and discussion

### Data preprocessing and statistical analysis for model development

**Figure 1:**
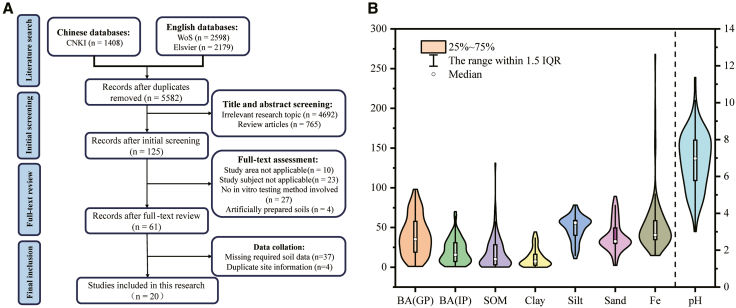
Overview of the dataset used for Cd bioaccessibility modeling (A) Literature screening workflow for Cd bioaccessibility data. (B) Violin plots with embedded boxplots showing the distributions of soil physicochemical properties collected from the selected studies. IQR refers to the interquartile range.

This study integrated soil Cd bioaccessibility data from multiple sites, which involved various *in vitro* testing methods. The detailed screening process is shown in Figure 1A. Previous studies have shown that although Cd bioaccessibility measured by different *in vitro* methods for the same samples is numerically comparable, statistically significant differences may still exist.^29^ To examine whether the dataset compiled in this study exhibits a similar pattern, the Kruskal-Wallis test was applied to compare results obtained from different methods. The results indicated that significant differences were also observed among methods (*p* < 0.01). Considering that the German Standard Institute method (DIN), the *in vitro* gastrointestinal method (IVG), and the simple bioaccessibility extraction test (SBET) are often used in combination with the physiologically based extraction test (PBET) or THE unified Bioaccessibility Research Group of Europe (BARGE) method (UBM)^24^ and that their stand-alone applications involve small sample sizes that are insufficient to support robust modeling, a Mann-Whitney U nonparametric test was further applied to compare the PBET and UBM methods. The results show that differences in bioaccessibility between the two methods were not significant (*p* > 0.05). Therefore, for sites involving multiple testing methods, this study consistently adopted results obtained using the PBET or UBM methods.

In the final dataset, PBET data accounted for 73.3%, UBM data for 15.8%, and other methods combined for 10.9%. The basic statistical characteristics of the integrated soil dataset are summarized in Table S5, and the distributions of the soil physicochemical properties are shown in Figure 1B. Substantial variability was observed across soil attributes: pH values were 3.01–11.37, soil organic matter (SOM) contents were 0.17–131.03 g/kg, and the proportions of clay, silt, and sand were 0.02%–44.43%, 10.82%–78.26%, and 2.30%–89.16%, respectively.

The Spearman correlation analysis results (Table 1) revealed a significant association between Cd bioaccessibility and its total concentration in soil. Gastric-phase bioaccessibility (BA(GP)) showed a highly significant positive correlation with total Cd, whereas intestinal-phase bioaccessibility (BA(IP)) was significantly negatively correlated. The latter may be attributed to the increased formation of insoluble Cd compounds or precipitates with anions in simulated intestinal fluid, which reduces Cd bioaccessibility.^30^ The BA(IP) exhibited a significant negative correlation with soil pH, implying that Cd release during the intestinal phase is strongly pH-dependent. Under lower pH conditions, an increase in positive surface charge weakens Cd adsorption and enhances its release into digestive fluid.^21^ The Fe content also had a significant negative correlation with BA(IP), possibly because Cd co-precipitates with Fe or is adsorbed by iron oxides in neutral intestinal environments, thereby suppressing dissolution. This explains why BA(IP) values were markedly lower than BA(GP) values.^13^^,^^31^ The BA(IP) was positively correlated with SOM. This may be attributed to the ability of organic matter to form soluble organometallic complexes with Cd, thereby enhancing its solubility.^32^ However, a previous study has also reported that carboxyl and hydroxyl functional groups in organic matter can adsorb Cd in soils.^33^ Soil texture also plays a role, with soils with a higher sand content, and therefore smaller surface areas and fewer adsorption sites, tending to facilitate Cd release into gastrointestinal fluid.^34^

**Table 1:** Spearman correlation analysis of Cd bioaccessibility

	pH	SOM	Clay	Silt	Sand	Fe	Cd
BA(GP)	0.03	0.03	−0.03	−0.22∗	0.29∗∗	−0.14	0.29∗∗
BA(IP)	−0.20∗	0.21∗	−0.09	−0.21∗	0.19	−0.21∗	−0.26∗

BA(GP) represents gastric-phase bioaccessibility; BA(IP) represents intestinal-phase bioaccessibility. “∗” indicates *p* < 0.05; “∗∗” indicates *p* < 0.01.

In this study, the correlations between Cd bioaccessibility and environmental factors were generally weak (|r| < 0.3), which is lower than those reported in some single-region studies. For example, Liu et al.^6^ reported a correlation coefficient of −0.40 between Cd bioaccessibility and clay content in mining-area soils, whereas Xie et al.^21^ reported a correlation coefficient of −0.44 between Cd bioaccessibility and soil pH in soils from mining and smelting areas. However, the dataset constructed in this study covers multiple regions and diverse soil types, resulting in substantial heterogeneity among samples (coefficient of variation [CV] = 23.17%–269.97%), which may weaken the overall linear correlation strength.^30^ Nevertheless, several significant correlations were still observed, indicating that these soil physicochemical properties exert non-negligible influences on Cd bioaccessibility.

### Performance comparison and validation of bioaccessible content prediction models

**Figure 2:**
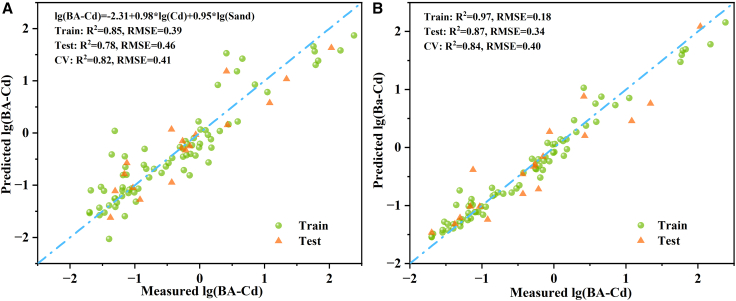
Comparison of Cd bioaccessible content prediction model performance (A and B) Stepwise regression prediction model; (B) random forest prediction model.

The bioaccessible content prediction model constructed using the stepwise regression approach is shown in Figure 2A. The final model retained only total Cd and sand fraction as predictors, indicating the absence of severe multicollinearity between them; other variables were excluded due to insufficient statistical significance (*p* > 0.05). The model exhibited a good fit for the training set (R^2^ = 0.85) and achieved an R^2^ value of 0.78 for the test set. The difference between the two (ΔR^2^ = 0.07) fell within an acceptable range, implying minimal overfitting and strong generalization capability. 5-fold cross-validation further supported this conclusion, as the cross-validated R^2^ value (CV R^2^ = 0.82) was close to that of the training set and higher than that of the test set, indicating consistent performance across data subsets and robust evaluation results.

The performance of the hyperparameter-optimized random forest model is shown in Figure 2B. The predicted and observed values were more tightly distributed around the 1:1 line, and both training and test R^2^ values were higher than those of the stepwise regression model, demonstrating the algorithm’s superior ability to capture complex nonlinear relationships. Although the test set R^2^ value (0.87) was slightly lower than that of the training set (0.97), it remained high, while the root-mean-square error (RMSE) remained low, indicating only mild overfitting. The cross-validated R^2^ value (0.84) closely matched the test set result, further confirming that the model maintained stable and superior predictive performance across different data subsets, with minimal sensitivity to random data partitioning. Overall, the random forest model demonstrated substantially higher predictive performance, both in terms of R^2^ and RMSE values, than the stepwise regression model.

**Figure 3:**
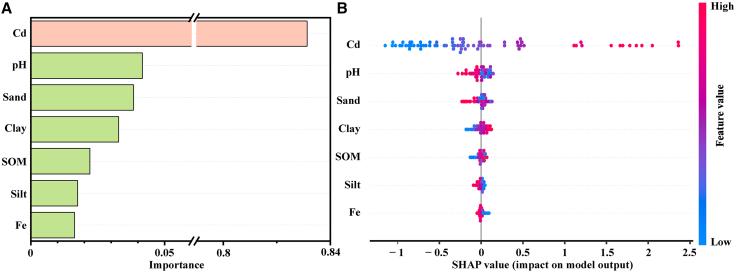
Feature importance and SHAP values for the random forest prediction model (A and B) Feature importance ranking of model predictors; (B) SHAP summary plot. The color indicates the feature value (red represents high values, and blue represents low values); the *x* axis shows the SHAP value, reflecting the direction and magnitude of each feature’s contribution to intestinal-phase Cd bioaccessibility.

Because the random forest model does not produce an explicit equation, this study employed feature importance analysis and SHapley Additive exPlanations (SHAP) values to enhance its interpretability. As shown in Figure 3, total Cd was the primary positive driving factor, accounting for 83.1% of feature importance. Soil pH was the second most influential feature, contributing negatively to the predicted values. Clay and sand represent opposing aspects of soil retention capacity. In soils with high clay and low sand contents, clay contributed negatively to the prediction, while sand exhibited either a negative or slightly positive effect, jointly reducing the predicted bioaccessible Cd content. A significant interaction effect was observed between Cd concentration and pH. In soils with low Cd concentrations (e.g., 0.40–0.49 mg/kg), the SHAP value of pH was −0.051 at pH 8.48 but 0.022 at pH 5, indicating that higher pH effectively reduced the predicted bioaccessible Cd content. However, in soils with high Cd concentrations (342 mg/kg), the SHAP value of pH was 0.032 at pH 7.03, indicating a positive contribution, which suggests that high Cd loading may mask the passivating effect of pH. Similarly, under comparable clay contents (36.5%–38.8%), when soil organic matter increased from 12.8 g/kg to 37 g/kg, the SHAP value of clay weakened from −0.182 to −0.142. This indicates that higher SOM levels reduced the negative contribution of clay, which may be related to the complexation of Cd by organic matter or competitive adsorption processes.^35^

**Figure 4:**
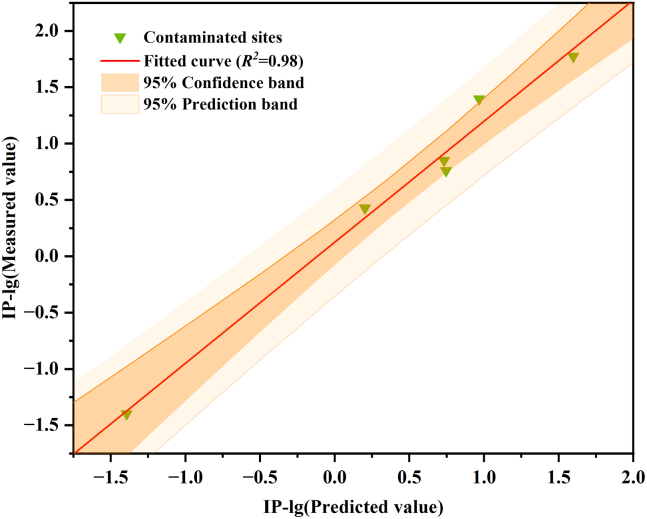
Contaminated sites verification results

The trained random forest prediction model was further validated using an independent validation dataset. The soil data from each validation site were input into the model to predict Cd bioaccessible content, and the predicted and observed values were compared to assess the model’s applicability under real-world conditions. As shown in Figure 4, all predicted values for the validation sites fell within the 95% confidence and prediction intervals, and the fitted curve achieved an R^2^ value of 0.98. These results indicate that the random forest-based prediction model demonstrated high accuracy and reliability in predicting soil Cd bioaccessibility under practical site conditions.

To more comprehensively evaluate the predictive performance of the proposed model, we conducted a systematic comparison with previously developed models for predicting soil Cd bioaccessibility. Most existing models are limited to single regions or specific soil types, such as mining areas, agricultural soils, or urban parks, and mainly rely on linear regression approaches.^36^^,^^37^^,^^38^^,^^39^ Although these models often perform well for the gastric phase, with R^2^ values typically ranging from 0.81 to 0.99, their predictive ability for (BA(IP)) varies considerably. For example, Liu et al.^6^ developed a stepwise regression model based on soils from a mining area in Guangxi, achieving an R^2^ of 0.93 for the gastric phase but only 0.77 for the intestinal phase. Similarly, Tian et al.^39^ developed multiple regression models for different land-use types in Dongguan, with model performance showing substantial variability (R^2^ = 0.28–0.88), and only the agricultural soil model exhibiting slightly higher performance than that of the present study. More recently, Xie et al.^21^ reported a random forest model with relatively high predictive accuracy (R^2^ = 0.83) based on mining soils from eight provinces in China; however, that model was restricted to (BA(GP)) and a single soil type.

In contrast, the model developed in this study is based on a composite dataset covering multiple regions and diverse soil types across China and specifically targets intestinal-phase Cd bioaccessible content using a random forest approach. The model demonstrates good generalization performance on the test set (R^2^ = 0.87). SHAP analysis further revealed nonlinear interactions among key controlling factors, such as the attenuation of pH effects under high Cd loading, providing mechanistic insights that linear models cannot capture. Moreover, because the intestinal phase represents a critical stage for contaminant absorption following oral exposure, the prediction target of this model is more directly aligned with the needs of human health risk assessment.

It should be noted that the model input parameters, including pH, soil texture, organic matter, and total Cd, are all routinely measured indicators in soil contamination investigations.^40^^,^^41^ In regional-scale risk assessments, once the random forest model has been trained, Cd bioaccessibility can be directly predicted using these existing data. This approach avoids the need to conduct time-consuming *in vitro* simulated gastrointestinal extraction tests for each individual sampling site, which typically require several hours, thereby enabling rapid predictions within seconds and significantly improving the efficiency of large-scale assessments involving numerous sampling sites.

Overall, the improved alignment of the prediction target with human absorption processes, the broader geographic coverage and diversity of soil types in the dataset, the enhanced interpretability of the model, and its rapid predictive capability based on routinely available data collectively represent clear advances over previous studies, providing a more reliable tool for region-specific health risk assessment of soil Cd contamination.

### National-scale spatial prediction of soil Cd bioaccessibility

**Figure 5:**
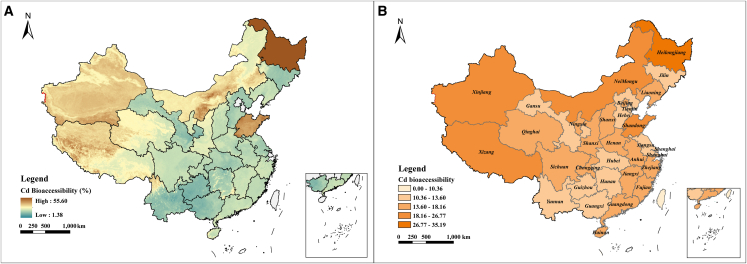
Spatial prediction of intestinal Cd bioaccessibility in soils across China (A and B) Spatial distribution of predicted Cd bioaccessibility across China (ordinary kriging interpolation; 1 km resolution); (B) provincial geometric mean of Cd bioaccessibility (31 provinces).

Based on nationwide soil property spatial data and the geometric mean Cd concentrations of each province, soil Cd bioaccessible content was predicted, from which Cd bioaccessibility was further calculated. The spatial distribution of Cd bioaccessibility is shown in Figure 5. The predicted bioaccessibility of soil Cd across China was 1.38%–55.60%, exhibiting a distinct “high in the north and low in the south” spatial pattern. Regions with relatively high Cd bioaccessibility were mainly distributed in northern provinces, such as Xinjiang, Heilongjiang, Jilin, and Inner Mongolia, where the geometric mean values were significantly higher than in other parts of the country. Notably, although previous studies have reported that soil Cd contamination in China generally follows a “higher in the southeast and lower in the northwest” pattern,^4^ our prediction results revealed an opposite trend in bioaccessibility. This indicates that total Cd is not the sole factor influencing Cd bioaccessibility.

The spatial variation in soil texture provides a physical explanation for this pattern (Figure S1). Compared with soils in northern China, soils in southern China generally contain a higher proportion of clay particles (Figure S1C), which enhances the adsorption and retention of Cd. In contrast, northern soils typically have a higher sand fraction (Figure S1E) and weaker retention capacity, making Cd more prone to release and migration. Although correlation analysis indicated a negative relationship between soil pH and Cd bioaccessibility, the overall bioaccessibility of Cd in the more alkaline northern soils remained higher than in the acidic southern soils. This indicates that Cd release is jointly influenced by soil texture, pH, and organic matter. For example, soils in Heilongjiang have high clay and low sand contents, but their relatively low pH and high SOM levels limit the fixation of Cd by clay minerals. Conversely, in Shandong soils, despite the high pH and low SOM, the high sand content dominates the Cd release behavior, resulting in similarly high bioaccessibility levels. Therefore, the elevated Cd bioaccessibility observed in northern soils could primarily be attributed to their weak retention capacity associated with sandy soil textures,^34^ and to the Cd species formed under alkaline conditions,^42^ which tend to be readily soluble but difficult to re-fix during gastrointestinal simulation processes.

In summary, the random forest model identified total Cd as the primary determinant in predicting bioaccessible Cd. This reflects that bioaccessible Cd, as a fraction of total Cd, is closely related to the overall level of soil Cd contamination. Meanwhile, soil physicochemical properties, including pH, texture, organic matter, and iron content, can further influence the model predictions by regulating the dissolution behavior of Cd under gastrointestinal conditions. Notably, at the national scale, the model-predicted bioaccessible Cd was substantially lower than the corresponding total Cd levels, indicating that only a portion of Cd (1.38%–55.60%) can be transformed into forms that are absorbable by the human body under gastrointestinal conditions. Therefore, using total Cd in health risk assessments may still lead to risk overestimation, whereas Cd bioaccessibility derived from model predictions can provide more accurate and reasonable risk estimates.

### Probabilistic health risk assessment based on Monte Carlo simulation

To obtain the probability distribution functions of soil Cd bioaccessibility across different geographical regions of China, the predicted results were first subjected to a normality test. The Q-Q plot revealed a significant deviation of the raw data from a normal distribution. After a natural logarithmic transformation, the data points aligned more closely with the theoretical line, indicating that the transformed data conformed to normality. Therefore, a log-normal distribution was fitted using IBM SPSS Statistics 26.0 to derive the probability distribution functions of Cd bioaccessibility for each region.

**Figure 6:**
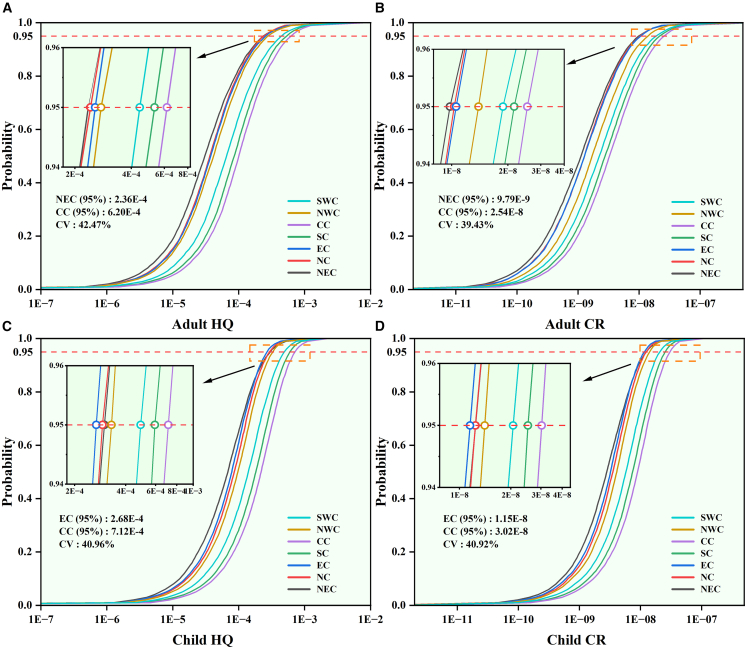
Regional probabilistic health risks of soil Cd after bioaccessibility adjustment (A–D) Adults non-carcinogenic risk; (B) adults carcinogenic risk; (C) children non-carcinogenic risk; (D) children carcinogenic risk. CV represents the coefficient of variation, and HQ and CR denote the total non-carcinogenic hazard quotient and carcinogenic risk for Cd in soil, respectively. The geographic region abbreviations represent Northwest China (NWC), North China (NC), Northeast China (NEC), Central China (CC), Southwest China (SWC), East China (EC), and South China (SC).

Based on soil Cd concentration data collected from 702 published studies, the geometric mean concentrations for each region were calculated to further evaluate the probabilistic carcinogenic risk (CR) and non-CR (hazard quotient, HQ) for both adults and children across the seven major geographical regions of China. Before bioaccessibility adjustment (Figure S4), no region exhibited a notable HQ, and the probability of adult CR exceeding the acceptable threshold was below 1%. The cumulative probability distributions of health risks after bioaccessibility adjustment are shown in Figure 6. Following correction, both the CR and HQ in all regions were well below acceptable thresholds, with CR values primarily ranging from 1 × 10^−9^ to 1 × 10^−8^ and HQ values ranging from 4 × 10^−5^ to 5 × 10^−4^. Compared with adults, the risk distributions for children shifted rightward and became more concentrated, indicating generally higher health risks and smaller interregional variability. After bioaccessibility adjustment, the 95^th^ percentile of health risk across regions decreased by 69.68%–86.90%, implying that bioaccessibility adjustment effectively prevents overestimation of risk and the resulting unnecessary remediation investment. The regional CVs for CR and HQ decreased from 52.00% to 62.41% to 39.42%–42.47%, indicating that the spatial heterogeneity of bioaccessibility substantially influences the uncertainty of health risk predictions.

**Figure 7:**
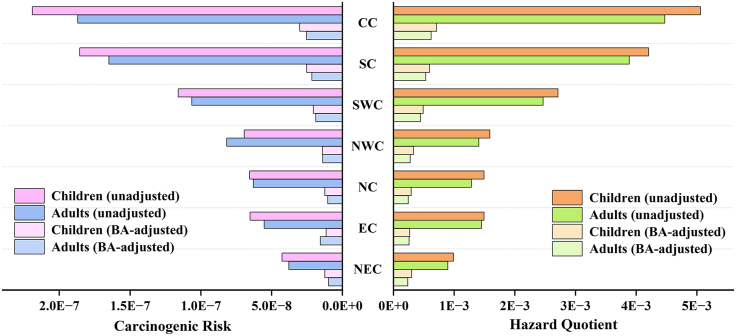
The 95th percentiles of CR and HQ for soil Cd across regions (A and B) Left, the 95th percentile of carcinogenic risk; (B) right, the 95th percentile of hazard quotient. Both are shown for adults and children before and after bioaccessibility adjustment.

The spatial comparison of 95^th^ percentile health risks before and after bioaccessibility adjustment is presented in Figure 7. A distinct spatial differentiation was observed, with CR and HQ sharing similar geographic patterns, i.e., highest in Central and South China, moderate in Southwest China, and lowest in Northwest, North, East, and Northeast China. Bioaccessibility adjustment did not alter the regional ranking of risks but markedly reduced their absolute differences. The higher Cd concentrations in southern China remained the dominant factor; however, bioaccessibility adjustment brought the actual risk levels closer to those in the north. Compared with adults, children showed higher CR and HQ values in most regions, primarily attributable to the higher ingestion rate of soil (IngR) and lower body weight (BW) assumed in the risk assessment parameters.^43^^,^^44^

### Sensitivity analysis for probabilistic health risk assessment

**Figure 8:**
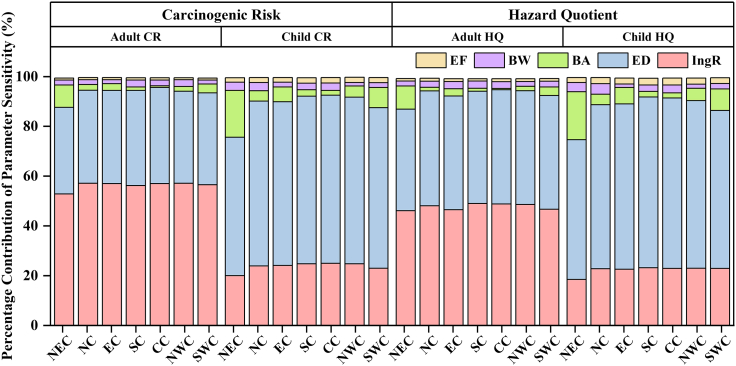
Parameter sensitivity analysis of the carcinogenic risk and hazard quotient for soil Cd across regions

The sensitivity analysis based on a Monte Carlo simulation identified spatial variations in the contribution rates of different exposure parameters to human health risk, thereby determining the key factors exerting the greatest influence on risk.^45^ The sensitivities of CR and HQ to soil Cd across regions, expressed as the absolute values of variance contributions, are presented in Figure 8. The parameters exerting the greatest influence on CR and HQ included IngR, exposure duration (ED), bioaccessibility, BW, and exposure frequency (EF). Among these, IngR exhibited the highest sensitivity for adults, contributing 52.8%–57.1% and 46.1%–49.0% of the contributions to CR and HQ, respectively. For children, ED showed the highest sensitivity, accounting for 55.6%–67.5% (CR) and 56.1%–68.6% (HQ). This difference is mainly attributed to the different exposure parameter settings for adults and children in the risk assessment model.^46^

The primary sources of uncertainty in soil Cd health risk were IngR and ED, while bioaccessibility ranked as the third most influential factor. Sensitivity analysis showed that the contribution of bioaccessibility to children’s risk models ranged from 1.9% to 19.3%, which was overall about 2-fold higher than that in adult models (0.5%–9.3%). This may stem from children’s lower BW and higher IngR, which amplify the influence of bioaccessibility variability and increase its proportional contribution. The spatial variation in bioaccessibility sensitivity corresponded to the north-south differentiation pattern of soil Cd bioaccessibility across China, with the highest sensitivity observed in northeastern regions, where bioaccessibility gradients varied most sharply.

Overall, both the probabilistic health risk assessment and the parameter sensitivity analysis highlighted the need to explicitly account for bioaccessibility in risk evaluation, with the effect being particularly critical for children. Given the pronounced geological heterogeneity across China, obtaining region-specific bioaccessibility measurements, especially in high-sensitivity areas such as Northeast China, is essential for improving the reliability of risk assessments. Therefore, conducting region-differentiated bioaccessibility studies holds substantial scientific relevance and management value, as such studies could effectively reduce the uncertainties in human health risk assessment.

### Conclusions and implications

This study developed a highly accurate and robust bioaccessible Cd content prediction model based on multi-source soil data. The model was applied to estimate Cd bioaccessibility across different geographical regions of China, followed by a probabilistic health risk assessment using a Monte Carlo simulation. The main conclusions were as follows: total Cd was the primary positive determinant, and significant nonlinear interactions existed among influencing factors. The random forest model outperformed stepwise regression in capturing these relationships and improving predictive accuracy. Soil Cd bioaccessibility generally exhibited a “higher in the north and lower in the south” pattern, with Cd release suppressed in the southern regions due to the combined fixation effects of clay, iron oxides, and organic matter. Under the regional-scale exposure assessment scenario adopted in this study, incorporating bioaccessibility correction did not alter the spatial ranking of health risks but reduced risk overestimation and narrowed interregional variability in risk estimates. Additionally, based on the Monte Carlo sensitivity analysis under the adopted exposure parameter distributions, bioaccessibility was identified as an important parameter following IngR and ED, with a greater contribution observed in children.

This study underscores the critical importance of incorporating bioaccessibility into health risk assessments and highlights the scientific and managerial significance of conducting region-specific bioaccessibility studies to reduce uncertainties in risk evaluation.

### Limitations of the study

In terms of data, *in vitro* simulation methods have not yet been fully standardized. Although data generated using the same method were preferentially used during model construction, methodological uncertainties may still exist. In addition, due to data availability constraints, several variables that may influence Cd bioaccessibility, such as climatic factors^47^ and Cd chemical speciation,^13^ were not included in the model. Regarding the model, although the random forest algorithm demonstrated strong predictive performance, its black-box nature limits the interpretation of the physicochemical mechanisms governing Cd bioaccessibility. The SHAP analysis provides insights into variable associations but cannot quantitatively describe causal relationships among the controlling factors. For spatial prediction, while the model reveals the macroscopic distribution pattern of Cd bioaccessibility across China, the use of provincial mean values may mask intra-provincial pollution hotspots and land-use differences. In risk assessment, although children were identified as the most vulnerable group, the analysis did not further differentiate among specific child age subgroups.

Future studies could address these limitations by incorporating more comprehensive datasets, including additional environmental control variables and higher-resolution Cd concentration data, as well as more refined exposure parameters. Furthermore, advanced approaches such as deep learning may be explored to capture more complex nonlinear relationships, thereby improving predictive accuracy and enhancing the resolution of risk assessment. In addition, the prediction model developed in this study could be further integrated into site risk assessment tools to support rapid screening and tiered management of contaminated sites.

## Resource availability

### Lead contact

Requests for further information and resources should be directed to and will be fulfilled by the lead contact, Youya Zhou (zhouyouya@tcare-mee.cn).

### Materials availability

The study did not generate new materials.

### Data and code availability

• This paper analyzes existing, publicly available data. The compiled and processed dataset supporting the findings of this study is accessible at https://doi.org/10.5281/zenodo.18171038.
• The codes used to generate the model are available in the following repository and are publicly accessible: https://doi.org/10.5281/zenodo.18080221.
• Any additional information required to reanalyze the data reported in this paper is available from the lead contact upon request.

## Acknowledgments

This study was supported by the 10.13039/501100012166National Key Research and Development Program of China (no.2023YFC3707703).

## Author contributions

Conceptualization, J.C., Y.Z. (Y.Z.); methodology, J.C., J.X., and C.Z.; investigation, formal analysis, visualization, writing − original draft, J.C.; writing − review & editing, supervision, Y.Z. (Y.Z.), and H.W.; funding acquisition, Y.Z. (Y.Z.), and J.X.; software and validation, C.Z. Y.Z. (Y.Z.), X.G., and Y.S.

## Declaration of interests

The authors declare no competing interests.

## STAR★Methods

### Key resources table

**Table:** 

REAGENT or RESOURCE	SOURCE	IDENTIFIER
**Deposited data**		

Compiled dataset	Zenodo	https://doi.org/10.5281/zenodo.18171038

**Software and algorithms**		

Python	Python Software Foundation	Python 3.12.0
scikit-learn	Python Software Foundation	scikit-learn 1.5.0
Statsmodels	Python Software Foundation	Statsmodels 0.14.2
ArcGIS	Esri	ArcGIS 10.8
Origin	OriginLab	Origin 2024
Oracle Crystal Ball	Oracle	Oracle Crystal Ball 11.1.3.0.0
SPSS	IBM	IBM SPSS Statistics 26.0

**Other**		

Code	Zenodo	https://doi.org/10.5281/zenodo.18080221

### Method details

#### Data collection and analysis for bioaccessibility

The data used for bioaccessibility modeling were collected from several databases, including the Web of Science, Elsevier Science Direct, and China National Knowledge Infrastructure (CNKI). The search keywords included “soil,” “site,” “heavy metal,” “cadmium,” and “bioaccessibility,” and Boolean operators were employed to construct the search expressions. The search covered literature published between 2000 and 2024. The inclusion criteria were: the study area was located in China; *in vitro* gastrointestinal simulation methods were employed, such as the physiologically based extraction test (PBET) and the unified Bioaccessibility Research Group of Europe (BARGE) method (UBM); the dataset included soil Cd total concentrations, bioaccessible contents, and relevant physicochemical properties; and the investigated soils were from actual contaminated sites rather than artificially prepared samples. The detailed screening process is shown in Figure 1A. After screening, in total 20 publications were included, yielding 195 site datasets that covered Cd total concentration, bioaccessible content, and soil parameters such as pH, soil organic matter (SOM), cation exchange capacity (CEC), iron (Fe) content, and the clay, silt, and sand fractions.

Data were organized and preliminarily analyzed using Microsoft Excel. For data presented in graphical form, numerical values were extracted using the Digitizer tool in Origin 2024. When only the total organic carbon (TOC) content was provided, the SOM content was estimated using a conversion factor of 0.58, as suggested by previous studies.^48^ During data preprocessing, the Kruskal-Wallis test was applied to examine overall differences among multiple groups, and the Mann-Whitney U test was used for pairwise comparisons between two groups. Subsequently, missing values were addressed using multiple imputation based on a random forest model. Outliers were identified and removed using IBM SPSS Statistics 26.0 based on Cook’s distance, with observations exceeding a threshold of D_i_ > 1 considered influential. Spearman’s rank correlation analysis was conducted to determine the relationships between soil characteristics and Cd bioaccessibility in the gastric and intestinal phases, with significance levels set at *p* < 0.05 and *p* < 0.01.

#### Integration of soil properties and spatial Cd data

To predict the spatial distribution of Cd bioaccessibility across China’s major geographical regions, nationwide soil property data, including pH, SOM, CEC, and the clay, sand, and silt fractions, were assembled. The data were obtained from the National Tibetan Plateau Data Center, which has integrated 8,979 soil profiles from the Second National Soil Survey,^16^ 1,540 profiles from the World Soil Information Service, 76 profiles from the First National Soil Survey, and 614 profiles from regional soil databases. Soil property data with a spatial resolution of 1 km × 1 km were used, and spatial processing was performed using ArcGIS 10.8 (the detailed workflow is provided in Methods S1). The soil Fe content data were obtained from *Background Values of Soil Elements in China*.^49^ Figure S1 shows the spatial distributions of soil properties in China.

Additionally, to assess the current status of soil Cd pollution, soil heavy metal data reported in publications over the past 5 years were systematically reviewed and Cd concentration data from topsoil (0−20 cm) samples were extracted to analyze the provincial-scale Cd pollution status and predict Cd bioaccessibility. The screening process excluded studies focused on localized contamination from accidental releases and those that did not provide explicit details on experimental methodology, sample processing, or the spatial extent of the study area. Ultimately, Cd concentration data covering various land-use types were extracted from 702 publications for subsequent analysis, and the provincial distribution of Cd exposure concentrations across China is illustrated in Figure S2.

#### Model development and validation

The prediction models for soil Cd bioaccessible content were developed using stepwise regression and random forest regression. Stepwise regression sequentially introduces candidate variables and evaluates the statistical significance of their partial regression coefficients at each step, while simultaneously examining whether pre-existing variables lose significance upon the inclusion of new predictors. Any variables failing to remain significant are removed accordingly.^50^ This procedure identifies the soil properties that exert a significant influence on Cd bioaccessible content. Random forest regression is based on the bootstrap aggregating principle, whereby multiple bootstrapped subsets are drawn with replacement from the training dataset. Each was used to build an individual decision tree, and the final prediction was generated by aggregating the outputs of all trees.^51^ The dataset was first log-transformed to approximate normality before modeling. All modeling procedures were performed in Python 3.12.0. Stepwise regression was implemented using the statsmodels library (version 0.14.2), and the random forest model was constructed with the RandomForestRegressor class in the scikit-learn library (version 1.5.0). The specific modeling process is shown in Figure S3.

During model development, the dataset was randomly divided into a training set (80%) and a test set (20%), and a grid search with five-fold cross-validation was performed to enhance model reliability and prevent overfitting.^19^ The optimal hyperparameters for the random forest model identified via grid search were: max_depth = none, max_features = 7, min_samples_leaf = 2, min_samples_split = 6, and n_estimators = 100. To enhance model interpretability further, feature importance rankings and SHapley Additive exPlanations (SHAP) values were generated to quantify the contribution of each variable to the prediction outcome.^52^^,^^53^ The SHAP values were derived from cooperative game theory and enabled a fair allocation of each feature’s contribution to the model prediction. Specifically, a SHAP value represents how much a feature’s value shifts the prediction relative to a baseline (mean prediction): positive values indicate an upward influence on the prediction, whereas negative values indicate the opposite.

After comparing the coefficient of determination (R^2^) and root-mean-square error (RMSE) across models,^20^ the best-performing model was applied to an independent validation dataset to assess its accuracy and robustness in real-world scenarios. The validation dataset included soil data from five provinces: Hubei, Fujian, Hunan, Jiangsu, and Liaoning (Table S1). The coefficients of variation ranged from 28.21% to 117.30%, indicating high spatial heterogeneity suitable for evaluating the model’s generalization capacity.

#### Probabilistic health risk assessment

##### Human health risk model

This study employed the health risk assessment model recommended by the United States Environmental Protection Agency (US EPA) to evaluate the health risks associated with Cd exposure quantitatively. Considering the differences in lifestyle and physiological characteristics between children and adults, population-specific exposure parameters were applied to separately assess carcinogenic and non-carcinogenic risks for the two groups. The results are intended to compare risk levels under different exposure scenarios, and the reported carcinogenic risk does not represent lifetime risk. The primary exposure pathways of heavy metals in soil include ingestion, inhalation, and dermal contact,^54^ and the corresponding risk values for each pathway were calculated using (Equation 1), (Equation 2), (Equation 3), (Equation 4), (Equation 5).^55^ Among these pathways, bioaccessibility was incorporated into the dose calculation for the ingestion pathway as a correction factor.

(Equation 1) $$\begin{array}{c} {ADD}_{ing}=\frac{C\times BA\times IngR\times EF\times ED}{BW\times AT} \end{array}$$

(Equation 2) $$\begin{array}{c} {ADD}_{inh}=\frac{C\times InhR\times EF\times ED}{PEF\times BW\times AT} \end{array}$$

(Equation 3) $$\begin{array}{c} {ADD}_{dermal}=\frac{C\times SA\times AF\times ABF\times EF\times ED}{BW\times AT}\times 10^{-6} \end{array}$$

(Equation 4) $$\begin{array}{c} HQ=\sum \frac{{ADD}_{i}}{{RfD}_{i}} \end{array}$$

(Equation 5) $$\begin{array}{c} CR=\sum {ADD}_{i}\times {SF}_{i} \end{array}$$

where ADD_ing_, ADD_inh_, and ADD_dermal_ represent the average daily doses (mg/(kg·d)) from ingestion, inhalation, and dermal contact, respectively. BA refers to the human bioaccessibility of contaminants in soil and is dimensionless. HQ and CR denote the total non-carcinogenic hazard quotient and carcinogenic risk index for Cd in soil, respectively. A non-carcinogenic risk is considered negligible when HQ < 1, and a carcinogenic risk is considered negligible when CR < 1 × 10^-6^. RfD_i_ and SF_i_ represent the reference dose and slope factor for Cd via the *i*^th^ exposure pathway, expressed in mg/(kg·d) and kg·d/mg, respectively.

Exposure parameter distributions, including BW (body weight), SA (skin surface area), and InhR (inhalation rate of soil), were obtained from the *Exposure Factors Handbook of Chinese Population (Adults)*^56^ and the *Highlight of Chinese Exposure Factors Handbook (Children)*^57^ for adults and children across China’s seven major geographical regions. The corresponding probability distribution functions for each parameter were fitted accordingly (Table S2). Detailed definitions and values of the exposure parameters (IngR, EF, ED, AT, PBF, AF, and ABF) are summarized in Tables S3, and the corresponding reference values for RfD_i_ and SF_i_ are listed in Tables S4.

##### Monte Carlo simulation

Monte Carlo simulations were performed using Oracle Crystal Ball 11.1.3.0.0, with 10,000 iterations conducted at a 95% confidence level to ensure the stability of the output results. Sensitivity analysis was subsequently performed to identify the major sources of uncertainty and to evaluate the contribution of each input parameter to the variability of the risk estimates.

### Quantification and statistical analysis

Statistical analyses were performed using Microsoft Excel, IBM SPSS Statistics 26.0, Origin 2024, and Python. Statistical details (e.g., sample size, p-values, and data presentation) are reported in the main text and in the figures and tables.

The raw dataset (n_1_ = 195) represents independent soil samples compiled from the literature and was analyzed using IBM SPSS Statistics 26.0. Differences among datasets obtained from different methods were evaluated using the Kruskal–Wallis test, and comparisons between PBET and UBM were conducted using the Mann–Whitney U test. After data screening, a refined dataset (n_2_ = 126) was used for subsequent modeling. Here, n denotes the number of independent samples. Spearman correlation analyses were performed using Origin 2024.

Model performance was evaluated using R^2^ and RMSE, and feature importance and interpretability were analyzed using SHAP, both implemented in Python 3.12.0. Spatial distribution maps (Figure 5A) were generated using the Kriging interpolation method within the Geostatistical Analyst module of ArcGIS 10.8. Probabilistic health risks were quantified using Monte Carlo simulations implemented in Oracle Crystal Ball 11.1.3.0.0, an add-in for Microsoft Excel. The simulations were performed with 10,000 iterations at a 95% confidence level, and the results were presented as probability distributions and visualized using Origin 2024.
