# Supplementary material for: Development of a soil cadmium bioaccessibility prediction model for human health risk assessment
Source: iScience. 2026 Apr 11;29(6):115679. doi: 10.1016/j.isci.2026.115679 (PMC13186039; doi:10.1016/j.isci.2026.115679)
Supplement: Document S1. Figures S1–S4, Tables S1–S5, and Methods S1 [file mmc1.pdf]

iScience, Volume *29*

Supplemental information

Development of a soil cadmium bioaccessibility prediction model for human health risk assessment

Jianghao Cao, Jie Xiong, Chaoyan Zhang, Ying Zhao, Xiaoxin Guo, Yimin Sang, Heming Wang, and Youya Zhou

List of Supplemental Information

Figure S1. Spatial distribution of soil properties in China.

Figure S2. Provincial distribution of Cd exposure concentrations across China.

Figure S3. Sample dataset and machine learning model development process.

Figure S4. Regional probabilistic health risks of soil Cd.

Table S1. Soil properties data of the independent validation set.

Table S2. Probability distribution functions of regionalized parameters in the health risk assessment model.

Table S3. Probability distribution functions of common parameters in the health risk assessment model.

Table S4. Reference dose and cancer slope factor for different exposure pathways of heavy metal Cd.

Table S5. Descriptive statistics of soil properties.

Methods S1. Spatial processing of soil property data and prediction workflow

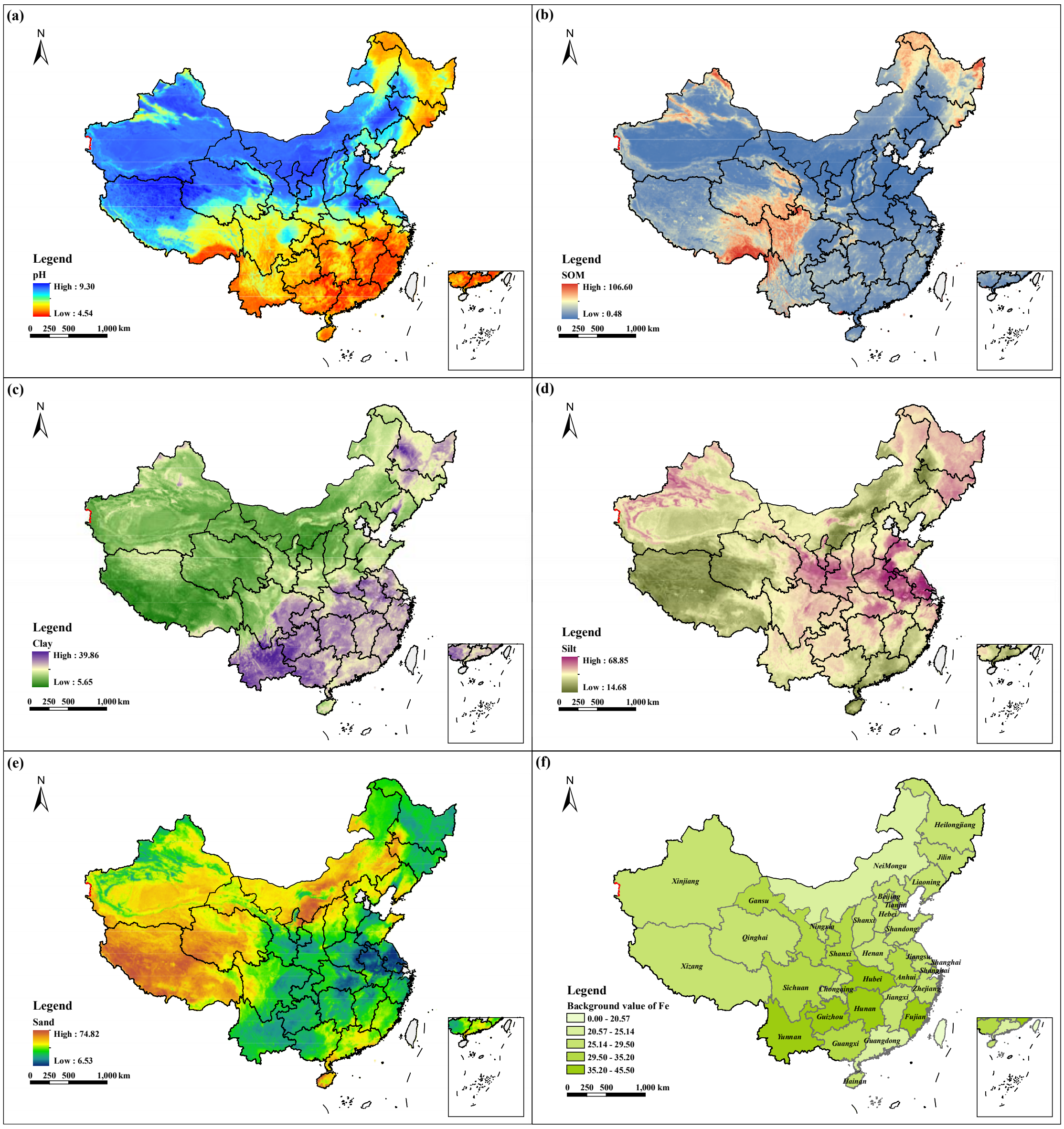

Figure S1. Spatial distribution of soil properties in China. (a) pH; (b) organic matter (g/kg); (c) clay fraction; (d) silt fraction; (e) sand fraction; (f) Fe content (g/kg)

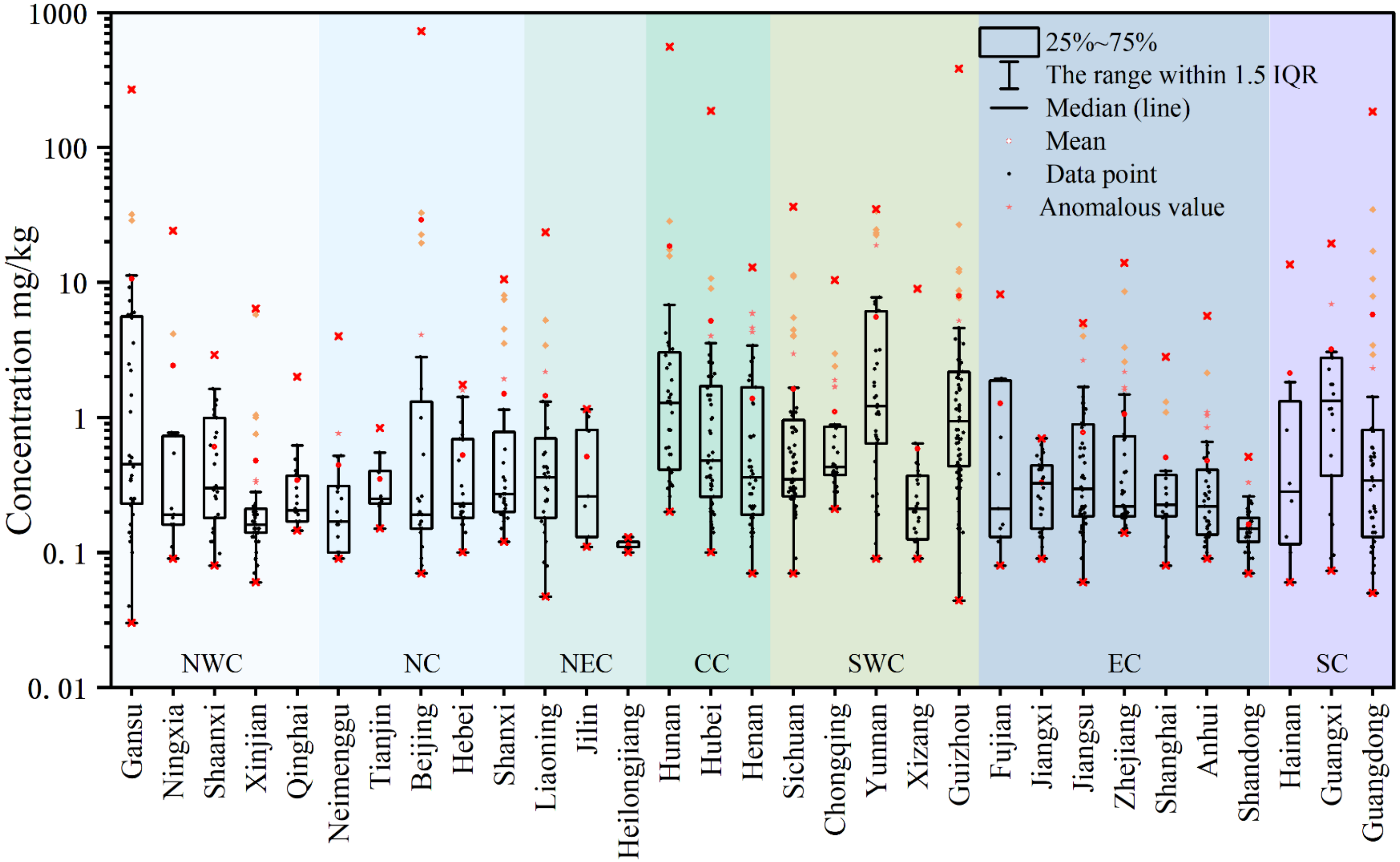

Figure S2. Provincial distribution of Cd exposure concentrations across China. The geographic region abbreviations represent Northwest China (NWC), North China (NC), Northeast China (NEC), Central China (CC), Southwest China (SWC), East China (EC), and South China (SC).

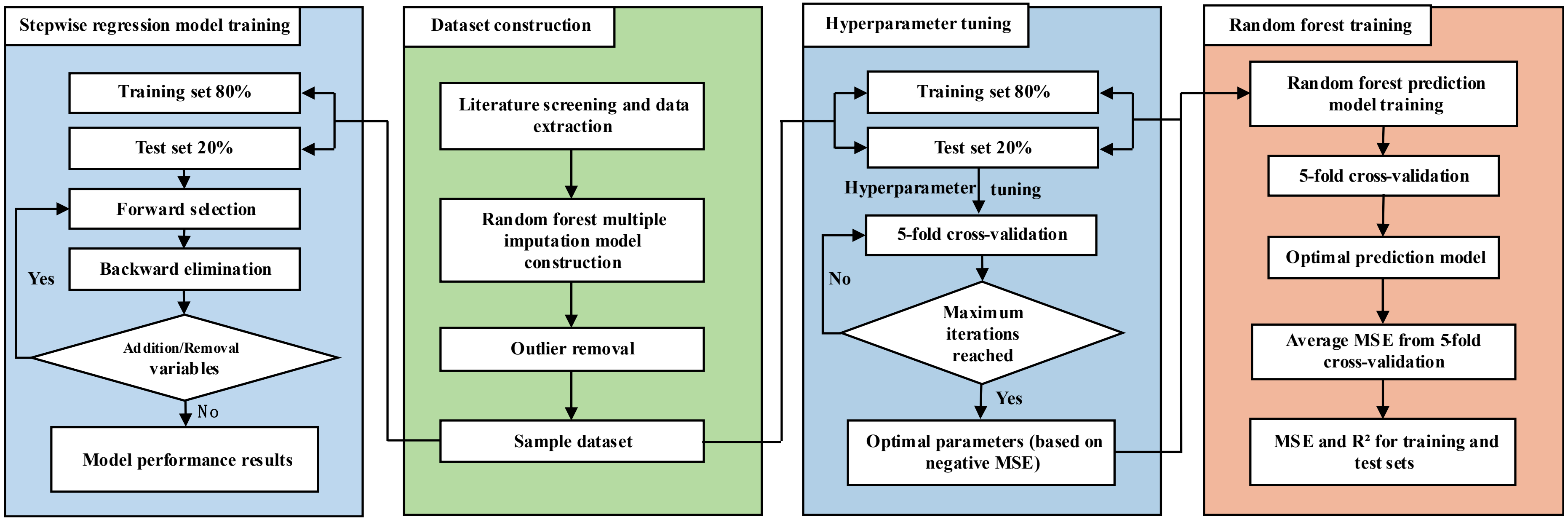

33

34 **Figure S3.** Sample dataset and machine learning model development process.

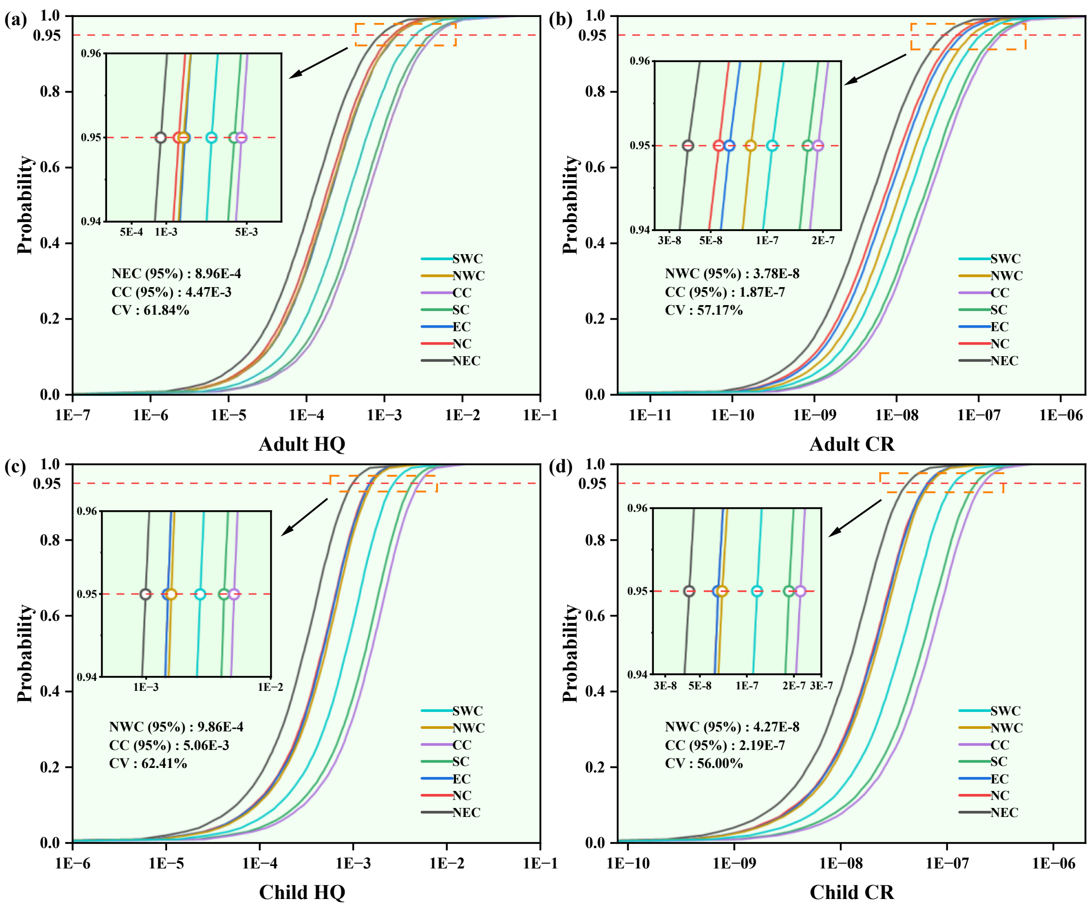

Figure S4. Regional probabilistic health risks of soil Cd.

Table S1 Soil properties data of the independent validation set.

Number	Cd/(mg/kg)	pH	SOM/(g/kg)	Clay/%	Silt/%	Sand/%	Fe/(g/kg)
1	244.4	7.21	12.16	44.43	25.16	30.41	65.88
2	17.45	7.82	1.90	29.55	39.59	30.86	58.63
3	36.10	8.34	30.63	7.27	58.89	31.37	160
4	67.30	7.05	30.06	10.98	58.01	31.37	34.2
5	129.00	3.01	9.24	5.81	57.73	32.70	32.3
6	32.49	5.83	30.30	11.70	57.40	31.06	58.52

Table S2 Probability distribution functions of regionalized parameters in the health risk assessment model.

Region	Child body weight (BW_c)	Adult body weight (BW_a)	Child skin surface area (SA_c)	Adult skin surface area (SA_a)	Child inhalation rate of soil ($InhR_c$)	Adult inhalation rate of soil ($InhR_a$)
	kg		m^2		m^3/d	
North China	N1(19.70,17.40)	N1(65.50,57.50)	N1(0.81,0.75)	N2(1.70,2.00)	N3(8.90,9.40)	N2(16.50,20.90)
Central China	N1(18.95,16.90)	N1(62.05,54.20)	N1(0.79,0.74)	N2(1.65,1.95)	N3(8.70,9.20)	N2(16.15,20.30)
South China	N1(18.20,16.40)	N1(58.60,50.90)	N1(0.77,0.72)	N2(1.60,1.90)	N3(8.50,9.00)	N2(15.80,19.70)
East China	N1(20.50,18.80)	N1(62.30,53.90)	N1(0.83,0.78)	N2(1.60,1.90)	N3(9.00,9.40)	N2(16.10,20.40)
Northwest China	N1(18.90,17.20)	N1(62.60,54.60)	N1(0.79,0.74)	N2(1.60,1.90)	N3(8.70,9.10)	N2(16.20,20.40)
Southwest China	N1(18.80,17.00)	N1(58.30,51.00)	N1(0.78,0.73)	N2(1.60,1.80)	N3(8.70,9.10)	N2(15.70,19.70)
Northeast China	N1(21.20,18.50)	N1(65.60,57.10)	N1(0.85,0.79)	N2(1.70,2.00)	N3(9.20,9.90)	N2(16.70,21.20)

Notes: a_1 and a_2 in $N_1(a_1, a_2)$ define the mean value and the 25th percentile for Normal Distribution 1; b_1 and b_2 in $N_2(b_1, b_2)$ define the mean value and the 95th percentile for Normal Distribution 2; and c_1 and c_2 in $N_3(c_1, c_2)$ define the mean value and the 75th percentile for Normal Distribution 3, respectively.

Table S3 Probability distribution functions of common parameters in the health risk assessment model.

Parameters	Unit	Potential distribution	Adult	Children	Reference
Exposure Frequency (EF)	day/year	Triangle	TR (180, 350, 365)		1
Exposure Duration (ED)	year	Uniform	UN(0,24)	UN(0,6)	2
Average Exposure Duration (AT)	day	Point	365*24 (non-carcinogenic)	365*6 (non-carcinogenic)	3
	day	Point	365*70 (carcinogenic)		3
Ingestion Rate of Soil (IngR)	mg/day	Log-normal	LN (50, 75)	LN (100, 50)	4,5
Particulate Matter Emission Factor (PEF)	m^3/kg	Point	1.36E+09	1.36E+09	2
Adherence Factor (AF)	mg/(cm^2·day)	Log-normal	LN (0.49, 0.54)	LN (0.65, 1.2)	6
Dermal Absorption Factor (ABF)	-	Point	0.01(carcinogenic)<br>0.001(non-carcinogenic)		3

Table S4 Reference dose and cancer slope factor for different exposure pathways of heavy metal Cd.[2]

Parameters	Unit	Ingestion	Inhalation	Dermal Contact
RfDi	mg/(kg·d)	1.00E-03	1.00E-05	1.00E-05
SFi	kg·d/mg	6.10E-01	6.30E+00	2.00E+01

Table S5. Descriptive statistics of soil properties.

Value	Cd (mg/kg)	BA(GP) %	BA(IP) %	pH	SOM (g/kg)	Clay %	Silt %	Sand %	Fe (g/kg)
Min	0.10	1.05	0.79	3.01	0.17	0.02	10.82	2.30	14.81
Max	1937.90	98.08	70.00	11.37	131.03	44.43	78.26	89.16	268.00
Mean	58.97	39.54	20.21	6.86	17.48	11.47	49.80	38.56	52.05
SD	217.53	24.36	15.26	1.53	19.84	11.33	15.41	18.86	34.04

Abbreviations: SD, standard deviation.

Methods S1. Spatial processing of soil property data and prediction workflow

To predict the spatial distribution of Cd bioaccessibility across major geographical regions of China, nationwide soil property data were collected, including soil pH, soil organic matter (SOM), cation exchange capacity (CEC), and particle-size fractions (Clay, Sand, and Silt). These data were obtained from the dataset "A China dataset of soil properties for land surface modeling (CSDLv2)" released by the National Tibetan Plateau Data Center, using the 1 km × 1 km resolution version.[7]

For model implementation and spatial data integration, a nationwide fishnet grid (5 km × 5 km) was generated in ArcGIS 10.8 and clipped by provincial boundaries. The Extract Multi Values to Points tool was then used to extract the 1 km resolution soil property data (pH, SOM, CEC, Clay, Sand, and Silt contents) to each grid point.

Based on the soil property attributes of each grid point, together with provincial background values of Fe and the geometric mean concentrations of Cd in each province, the trained random forest model was applied to predict Cd bioaccessible concentrations and calculate the corresponding bioaccessibility. The predicted values were subsequently assigned to the corresponding grid points. Finally, ordinary kriging interpolation was used to generate a continuous national prediction surface. The spatial reference system was consistent with that of the soil property dataset, and the final output resolution was 1 km.

Supplemental references:

1. Sun, J., Zhao, M., Huang, J., Liu, Y., Wu, Y., Cai, B., Han, Z., Huang, H., and Fan, Z. (2022). Determination of priority control factors for the management of soil trace metal(loid)s based on source-oriented health risk assessment. J. Hazard. Mater. *423*, 127116. https://doi.org/10.1016/j.jhazmat.2021.127116.
2. Lei, M., Li, K., Guo, G., and Ju, T. (2022). Source-specific health risks apportionment of soil potential toxicity elements combining multiple receptor models with Monte Carlo simulation. Sci. Total Environ. *817*, 152899. https://doi.org/10.1016/j.scitotenv.2021.152899.
3. Yuan, B., Cao, H., Du, P., Ren, J., Chen, J., Zhang, H., Zhang, Y., and Luo, H. (2023). Source-oriented probabilistic health risk assessment of soil potentially toxic elements in a typical mining city. J. Hazard. Mater. *443*, 130222. https://doi.org/10.1016/j.jhazmat.2022.130222.
4. Duan, X. (2016). Highlight of Chinese Exposure Factors handbook (Children) (China Environment Publishing Group).
5. Ministry of Environmental Protection. (2013). Exposure Factors Handbook of Chinese Population (Adults) (China Environment Publishing Group).
6. Shi, J., Du, P., Luo, H., Wu, H., Zhang, Y., Chen, J., Wu, M., Xu, G., and Gao, H. (2022). Soil contamination with cadmium and potential risk around various mines in China during 2000-2020. J. Environ. Manag. *310*, 114509. https://doi.org/10.1016/j.jenvman.2022.114509.
7. Shi, G., and Shangguan, W. (2025). A China dataset of soil properties for land surface modeling (version 2, CSDLv2). (National Tibetan Plateau Data Center). https://doi.org/10.11888/Terre.tpdc.301235.
